# Meta-studies in land use science: Current coverage and prospects

**DOI:** 10.1007/s13280-015-0699-8

**Published:** 2015-09-25

**Authors:** Jasper van Vliet, Nicholas R. Magliocca, Bianka Büchner, Elizabeth Cook, José M. Rey Benayas, Erle C. Ellis, Andreas Heinimann, Eric Keys, Tien Ming Lee, Jianguo Liu, Ole Mertz, Patrick Meyfroidt, Mark Moritz, Christopher Poeplau, Brian E. Robinson, Ralf Seppelt, Karen C. Seto, Peter H. Verburg

**Affiliations:** Environmental Geography Group, VU University Amsterdam, De Boelenlaan 1087, 1081 HV Amsterdam, The Netherlands; University of Maryland at Baltimore County, 211 Sondheim Hall, 1000 Hilltop Circle, Baltimore, MD 21250 USA; National Socio-Environmental Synthesis Center, 1 Park Place, Suite 300, Annapolis, MD USA; School of Life Sciences, Arizona State University, Tempe, USA; Department of Life Sciences, University of Alcalá, 28805 Alcalá de Henares, Spain; Centre for Development and Environment, University of Bern, Bern, Switzerland; Department of Geography, University of Florida, Gainesville, USA; Earth Institute, Columbia University, New York, USA; Center for Systems Integration and Sustainability, Department of Fisheries and Wildlife, Michigan State University, 115 Manly Miles Building, East Lansing, MI 48824 USA; Department of Geosciences and Natural Resource Management, University of Copenhagen, Øster Voldgade 10, 1350 Copenhagen K, Denmark; F.R.S.-FNRS & Georges Lemaître Centre for Earth and Climate Research, Earth and Life Institute, Université Catholique de Louvain, Place Pasteur 3, bte L4.03.08, 1348 Louvain-la-Neuve, Belgium; Department of Anthropology and Environmental Science Graduate Program, The Ohio State University, 174 W. 18th Avenue, Columbus, OH 43210 USA; Department of Ecology, Swedish University of Agricultural Sciences (SLU), Ulls Väg 16, Box 7044, Uppsala, Sweden; Thuenen Institute of Climate-Smart Agriculture, Braunschweig, Germany; Department of Geography, McGill University, 805 Sherbrooke Street W, Montreal, QC H3A 0B9 Canada; Department of Computational Landscape Ecology, UFZ - Helmholtz Centre for Environment Research, Permoserstraße 15, 04318 Leipzig, Germany; Yale School of Forestry and Environmental Studies, Yale University, 195 Prospect Street, New Haven, CT 06511 USA; Instituto de Ciencias Ambientales y Evolutivas, Avenida Rector Eduardo Morales Miranda, Edificio Pugín, Universidad Austral de Chile, Valdivia, Chile; Department of Social Science and Policy Studies, Worcester Polytechnic Institute, Worcester, MA 01609 USA

**Keywords:** Land use change, Human–environmental systems, Drivers, Impacts, Meta-analysis, Systematic review

## Abstract

Land use science has traditionally used case-study approaches for in-depth investigation of land use change processes and impacts. Meta-studies synthesize findings across case-study evidence to identify general patterns. In this paper, we provide a review of meta-studies in land use science. Various meta-studies have been conducted, which synthesize deforestation and agricultural land use change processes, while other important changes, such as urbanization, wetland conversion, and grassland dynamics have hardly been addressed. Meta-studies of land use change impacts focus mostly on biodiversity and biogeochemical cycles, while meta-studies of socioeconomic consequences are rare. Land use change processes and land use change impacts are generally addressed in isolation, while only few studies considered trajectories of drivers through changes to their impacts and their potential feedbacks. We provide a conceptual framework for linking meta-studies of land use change processes and impacts for the analysis of coupled human–environmental systems. Moreover, we provide suggestions for combining meta-studies of different land use change processes to develop a more integrated theory of land use change, and for combining meta-studies of land use change impacts to identify tradeoffs between different impacts. Land use science can benefit from an improved conceptualization of land use change processes and their impacts, and from new methods that combine meta-study findings to advance our understanding of human–environmental systems.

**Electronic supplementary material:**

The online version of this article (doi:10.1007/s13280-015-0699-8) contains supplementary material, which is available to authorized users.

## Introduction

Land use science aims to understand how and why land use changes and what the impacts of these changes are (Rindfuss et al. 2008). Land use change processes and their impacts have often been studied using case studies. In this paper, we refer to a *case study* as primary research rooted in a particular place and context. Case studies typically explore how a particular constellation of drivers leads to observed land use changes, or how a particular land use change results in impacts in a specific location. A case-study approach allows for a detailed analysis, which is required to gain expertise in these processes (Flyvbjerg 2006). At the same time, the validity of case-study results is inherently limited to the particular historical and geographic contexts of the case, and cannot be generalized.

Various publications have synthesized findings from individual case studies in order to generalize our understanding of land use change processes and their impacts. We refer to these as *meta*-*studies*. These meta-studies are frequently conducted to build or advance theories, to identify further research needs, and to evaluate or inform policy making (Magliocca et al. 2015b).

In contrast with the term meta-analysis, meta-studies do not necessarily imply rigorous statistical treatment of case-study evidence. Such statistical treatment is frequently not possible in land use science due to the complexity of empirical case studies, differences in case-study design, and the preponderance of qualitative results included in case studies. Hence, the term meta-studies includes meta-analyses, systematic reviews, and other secondary studies that aim to synthesize case-study findings. Meta-studies have, for instance, addressed urbanization (Seto et al. 2011), agricultural intensification (Keys and McConnell 2005), and wetland conversion (van Asselen et al. 2013). A larger number of meta-studies have synthesized the various types of impacts of land use changes, such as dynamics in soil organic carbon (Poeplau et al. 2011), changes in biodiversity (Gibson et al. 2011), and consequences for ecosystem services in general (Seppelt et al. 2011).

Comparison of case studies inevitably requires fitting each case in a common framework, both from a methodological point of view—e.g., regarding quantitative or qualitative variables included in the analysis—and from a conceptual point of view. However, land use change processes and their impacts are best seen as complex socioecological systems with multiple components which interact over space and time, and have feedback mechanisms and chains of causation (Verburg 2006; Claessens et al. 2009). Consequently, it is a challenge to structure these complex causal chains within the simplifying framework of meta-studies. Here, our objective is to assess and understand to what extent the combined results of the existing meta-studies shed light on the full causal chain linking underlying drivers to proximate causes to land use change to their impacts. Based on this review, we provide suggestions for improving these conceptualizations, or overcome the limitations that are inherent to this.

## Challenges for conducting meta-studies in land use science

Meta-studies are secondary studies that synthesize empirical, case-based, research in order to identify commonalities and differences through a systematic and structured analysis. Synthesizing information from primary studies in land use science is often not straightforward for several reasons:

- Land use changes and their impacts are usually studied from real-world observations rather than experiments in a controlled environment. Consequently, climate and other biophysical conditions, policy context, events, local culture, and social constructs may vary across case-study sites and are difficult to control for in research design. These differences in local conditions can cause confusion between correlation and causation, and restrict the comparability of case-study findings (Bowler et al. 2012). Especially for social sciences, it has been argued that predictive theories cannot be found due to the idiosyncrasies of case studies (Flyvbjerg 2006). In such situations, generalization is at best limited to a qualitative level.
- The variable of interest cannot always be expressed in a single indicator such as monetary units or agricultural yields. Some impacts of land use change can be quantified using a scalar indicator, such as species richness in biodiversity assessments (Letourneau et al. 2011). However, other biodiversity indicators might respond differently to similar changes (Chiarucci et al. 2011). The situation is even more complex for land use change processes, as these are not always quantifiable, and drivers of land use change are often reported in a narrative format, which seriously constrains a systematic analysis (Keys and McConnell 2005).
- Empirical evidence for land use change processes and impacts is collected and analyzed by researchers from different disciplines, including ecology, geography, economics, and sociology. Therefore, data sources and research methods in different case studies might not, or only partially, overlap. The diversity in data and methods ranges from qualitative interviews (e.g., Sutherland (2012)) to the statistical analysis of spatial data (e.g., Prishchepov et al. (2013)). Moreover, disciplinary backgrounds can influence the independent variables that are considered for investigation. Consequently, only few studies include a comprehensive coverage of socioeconomic and biophysical drivers (Hersperger and Bürgi 2009).
- Case studies strongly differ in their spatial scale. For example, cases included in the meta-study of wetland conversion by van Asselen et al. (2013) range from 1 to 150 000 km^2^. As land use change processes and impacts are scale dependent, the comparison of case studies conducted at different scales may become troublesome (Veldkamp et al. 2001). Neglecting these differences might lead to bias due to aggregation issues and misinterpreted scale effects (Coleman 1990). Two well-documented scale effect are the modifiable area unit problem, in which statistical results can depend on the definition of spatial units in which a variable is aggregated (Openshaw 1983), and the ecological fallacy, in which inferences about the nature of individual cases is incorrectly deduced from inference of the group (Robinson 1950).
- The time period of the case studies can also vary substantially. A recent review of agricultural land use change processes in Europe (van Vliet et al. 2015) included study periods from 3 to 61 years, which were not always overlapping. Biophysical and socioeconomic conditions are changing considerably over time, e.g. through climate change or fluctuations in the world economy. In addition, the duration between observations affects the results as some processes require more time to manifest themselves or have a time lag. These temporal issues might limit the comparability of cases.
- The representativeness of meta-study results depends on the distribution of case-study locations, which are beyond the control of the meta-study design. Therefore, meta-studies are vulnerable to the sampling effect (Koricheva et al. 2013). Powers et al. (2011) for instance show that locations of field observations of soil organic carbon stocks in the tropics are neither representative of the tropics nor of locations that have undergone land cover conversion. Similarly, Seto et al. (2011) find many urban expansion studies in the USA and China, but some of the largest cities by population and size have not been studied, suggesting biases in the selection of case-study locations. Consequently, meta-studies are not necessarily representative syntheses of specific land use change processes or impacts. With extreme sampling biases, meta-studies only indicate what processes and locations have been studied extensively, but provide little information about these processes.

## Materials and methods

We systematically searched in ISI Web of Science for all meta-studies that analyze land use change processes or land use change impacts, building on the review by Magliocca et al. (2015b). Meta-studies were defined as studies that are secondary studies, i.e., based on other previously published primary research, and that are systematic in their analysis, i.e., excluding literature reviews in which the translation between cases and the meta-study findings was not tractable. Land use change includes land cover changes caused by land use changes, but excludes studies in which humans are not the direct driver of land-cover change, such as the impact of climate change on vegetation dynamics. We further restricted ourselves to studies that are either based on observed changes (before/after), or studies that compared multiple different land uses (using space–time substitution). Only studies were selected that focus on the landscape scale, e.g. excluding plot level or even laboratory studies that are frequently used in agronomy. A more detailed description of the selection criteria, the search procedure, and the search terms is provided in the 10.1007/s13280-015-0699-8.

Meta-studies were coded for their regional coverage, the number of primary studies included, the number of observations included, and the synthesis method. Regional coverage indicates the spatial extent of cases included in a specific meta-study, which was not subdivided in predefined regions but based on the description of the original authors. A number of primary studies and a number of observations were taken from the meta-study or its supplementary material. Observations are defined here as the unit of analysis that is included in the meta-study. Synthesis methods are based on the classification used by Magliocca et al. (2015b). In this paper, we do not extensively discuss meta-study methods themselves, as this topic has been discussed elaborately by Magliocca et al. (2015b).

We distinguished between the following major land use types: agricultural land (all land that is mainly used for agricultural production, including croplands, managed pastures and agroforestry), forest (also including woodlands), grassland (excluding managed pastures, but including savannas, as well as seminatural land used for grazing and pastoralism), wetlands, urban land, and multiple land uses. Studies that focus on conversion from one class into another were coded for the land use type that the paper focuses on. In some studies, this is the land cover that is converted, for example, wetland conversion (van Asselen et al. 2013), while in other cases, this is the land use into which the land is converted, such as forest restoration (Rey Benayas et al. 2009). While this classification ignores the other land use types that are inherently included in a land use change, it provides a clearer picture of the research focus of the studies included. Studies that focus on more than one major land use type were coded as “multiple land uses.”

Meta-studies of land use change processes were further analyzed for the land use change process that was analyzed, and the conceptualization of this land use change process. Meta-studies of land use change impacts were also coded for the specific consequence addressed, in addition to those variables used for meta-studies of land change processes. Land use change processes and consequences were only described qualitatively, due to the wide range of processes, consequences, and conceptualizations, which was found in these studies.

## Meta-study coverage of land use change processes and their impacts

The systematic search yielded 5296 publications from which 138 were selected for this study based on the eligibility criteria. Of these studies, 20 meta-studies analyze land use change processes, while 118 meta-studies analyze impacts of land use changes. For interpretation, we divided the latter group in impacts on biodiversity (*n* = 59), biogeochemical cycles (*n* = 33), hydrology (*n* = 15), food production (*n* = 7), and socioeconomic impacts (*n* = 4). These groups are not strictly delineated but nevertheless reflect the main topics covered by these meta-studies.

**Fig. 1:**
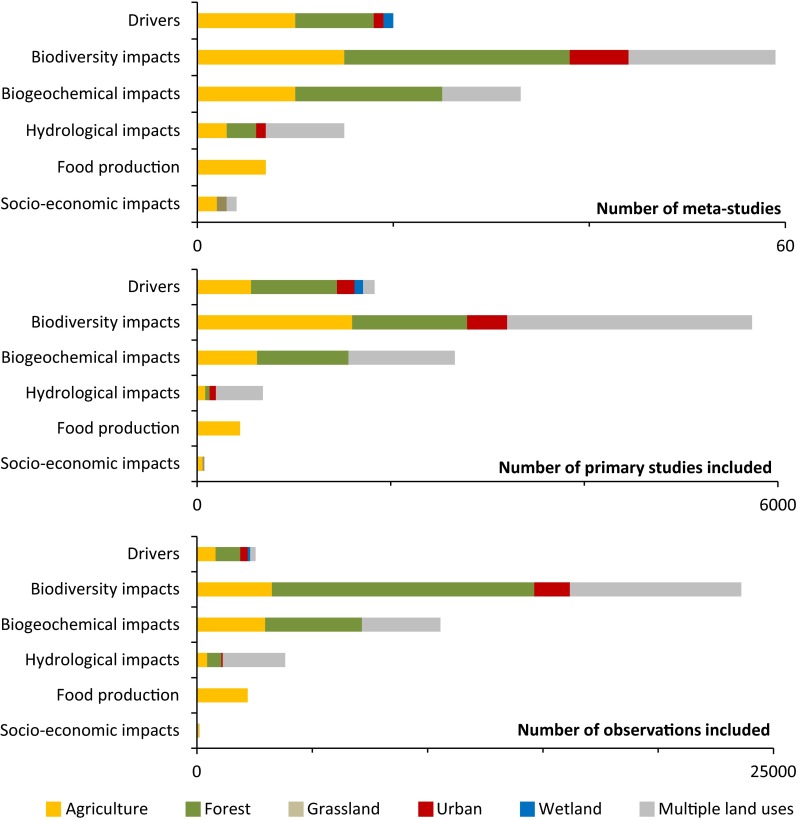
Distribution of meta-studies included in this review, and the primary studies and observations these meta-studies are based on

All meta-studies combined are based on 11 429 primary studies, and 42 840 observations. The number of observations per meta-study is divided unevenly, which can at least partly be explained by the nature of these observations. Observations on land use change processes typically comprise a complete case study, requiring relatively many resources. Consequently, primary studies typically report only one or a limited number of case studies in one paper, which explains the relatively low number of observations per primary study in meta-studies of land use change processes (e.g., van Asselen et al. 2013; van Vliet et al. 2015). Primary studies of biodiversity or biogeochemical cycles, on the other hand, often include multiple observations from one study site, for instance, by sampling multiple taxa in one location or sampling different plots in one study site. Therefore, meta-studies of biodiversity impacts of land use change (De Frenne et al. 2011; Mantyka-pringle et al. 2012) and biogeochemical impacts of land use change (Ogle et al. 2012; Bonner et al. 2013) sometimes include a relatively large number of observations. Figure 1 shows the distribution of land uses over meta-studies and the primary studies underlying these meta-studies. Details of the individual meta-studies are presented in Tables 10.1007/s13280-015-0699-8–10.1007/s13280-015-0699-8.

The difference between the number of meta-studies that address land use change processes and those that address various land use change impacts can be explained by the number of primary studies available, the nature of the synthesis, and the type of data that is provided in primary data sources. The number of primary studies indicates the available base material for meta-studies, which is particularly high not only for biodiversity impacts, and biogeochemical cycles, but also for observations of the land use change processes themselves. The nature of the synthesis and the type of available data are strongly related. Primary studies in biodiversity and biogeochemical cycles as well as food production studies typically yield a quantitative measurement, such as species abundance, soil organic carbon content, or crop yield per hectare. These types of results can be used for a quantitative analysis, including meta-analysis of effect size. Studies on land use change processes and socioeconomic impacts often yield a qualitative or semiquantitative result. Synthesizing these types of primary studies is much less straightforward, and consequently there are relatively fewer of those meta-studies.

The uneven distribution of meta-studies across land uses might reflect a sampling effect caused by the availability of case studies, and potentially the underlying funding priorities (Seppelt et al. 2011). In land use science, originally much attention was given to conversion of tropical forests, while processes like wetland conversion and grassland changes have attracted less attention (Verburg et al. 2011).

### Meta-studies of land use change processes

Studies that assess drivers of land use change predominantly assess changes in one specific land use type, such as urbanization, wetlands conversion, or agriculture change, although frequently, this includes conversion from or into another land use type. Not all land uses have received equal attention, as shown in Table 10.1007/s13280-015-0699-8. Eight meta-studies focus on forest changes, and ten address agricultural land use change, while other land use changes, like urban expansion (Seto et al. 2011) and wetland conversion (van Asselen et al. 2013) have been studied only once. No meta-study thus far has focused on grassland changes specifically, although there are a number of papers that synthesize theoretical and practical issues concerning pastoral land use in different world regions (Galvin 2009; Behnke et al. 2011; Moritz et al. 2013; Sayre et al. 2013). These studies analyze changes in land tenure, privatization and enclosure, fragmentation of rangelands, and population pressure on natural resources, albeit mostly in a qualitative way.

Land use change includes land cover conversion as well as changes in land management. Rounsevell et al. (2012) indicate that by far, the largest share of the increase in grain production in Europe in the last half century has been achieved through intensifying land management (including irrigation, mechanization, the introduction of new cultivars, and increased fertilizer usage). In contrast, the cropland area in Europe has decreased slightly in the same period. However, land cover and land management changes have not received equal attention in meta-studies. For instance, all eight meta-studies on forest change assess drivers for losses in forest cover, while only one addresses forest degradation. Similarly, urbanization is only studied in terms of land cover change, while more subtle land use changes such as peri-urbanization have not been analyzed in meta-studies yet. On the other hand, six out of nine meta-studies on agricultural land use also assess land management changes. The emphasis on land cover conversion reflects a focus of land use science on the more dramatic land cover changes which can be observed based on remote sensing imagery.

### Meta-studies of land use change impacts

Meta-studies of impacts of land use change are more abundant than studies of land use change processes and cover a wide range of land use change impacts, including biodiversity, biogeochemical cycles, hydrologic impacts, food production, and socioeconomic impacts. Other ecosystem services were included in several studies, mostly in combination with biodiversity (Rey Benayas et al. 2009; Kennedy et al. 2013), but not as a separate meta-study. Note that these groups are not strictly defined, but instead introduced to provide an overview of the topics covered by these meta-studies. Meta-studies that assess impacts on biodiversity, species richness or related indicators are dominant (59 meta-studies, see Table 10.1007/s13280-015-0699-8). Effects of land use, and land use change on biogeochemical cycling have been studied in 33 meta-studies (Table 10.1007/s13280-015-0699-8). Considerably fewer studies are available for land use change impacts on hydrology (15 meta-studies, Table 10.1007/s13280-015-0699-8), food production (7 studies, Table 10.1007/s13280-015-0699-8), and socioeconomic impacts (4 meta-studies, Table 10.1007/s13280-015-0699-8). It should be noted, however, that several meta-studies on food production did not meet our criteria, because they focused on biophysical processes alone, such as climate change, or because results were not applicable at a landscape scale.

The number of primary studies and the number of observations included in meta-studies of land use change impacts varies considerably. Meta-studies of biodiversity impacts and impacts on biogeochemical cycles are based on a high number of primary studies (on average 92 and 81, respectively). On the other hand, meta-studies on socioeconomic impacts only have an average of 19 primary studies per meta-study. Socioeconomic impacts of land use change, such as livelihoods, are not easily expressed in one comparable and quantitative measure, which hampers meta-study synthesis (Muchena et al. 2005; Cramb et al. 2009).

Tables 10.1007/s13280-015-0699-8, 10.1007/s13280-015-0699-8, 10.1007/s13280-015-0699-8, 10.1007/s13280-015-0699-8, and 10.1007/s13280-015-0699-8 show that the majority of the studies focus on impacts of changes in agriculture and forests, as these are included in 37 and 41 studies, respectively. On the other hand, impacts of urbanization and grassland dynamics have been investigated in only 8 and 1 studies, respectively. Impacts of wetland conversions have not been synthesized at all. 32 studies do not focus on one land use or land use change specifically, but instead analyze the consequences related to changes in multiple land uses. While most studies on multiple land uses include agriculture and forest, grasslands are also well represented here (see, e.g., Kim and Jackson 2012; Montero-Castaño and Vilà 2012). Meta-studies of land use change impacts analyze consequences of land cover conversions, such as farmland abandonment (Queiroz et al. 2014) as well as more subtle land intensity changes, such as different forest management regimes (Holloway and Smith 2011; Schmidt et al. 2011).

## Patterns and prospects for meta-studies in land use science

### Conceptualization of land use change processes

Before the first meta-studies in land use science were conducted, syntheses of land use change processes came from expert knowledge, often based on insights from case-study research. Lambin et al. (2001) provide a seminal synthesis of driving factors for different land use changes based on an expert workshop. Not surprisingly, findings were later confirmed by meta-studies that provided a structured synthesis of case-study evidence. For example, Lambin et al. (2001) suggest that multiple pathways of agricultural intensification exist, and different driver combinations as well as the possibilities for import of agricultural products are identified as major drivers. These findings have been confirmed by meta-studies of agricultural intensification in the tropics (Keys and McConnell 2005) and in Europe (van Vliet et al. 2015). Both studies identify multiple pathways of intensification and find that globalization and teleconnections through the trade system are important drivers of agricultural land use change. The advantage of meta-studies over expert knowledge is that they allow quantifying the occurrence of different drivers of land use change, thereby indicating their relative importance. On the other hand, there is a limit to which especially socioeconomic processes can be generalized (Flyvbjerg 2006), and therefore, qualitative reviews will remain relevant for synthesizing land use change processes that are not easily captured in coding schemes of structured, quantitative, meta-studies.

**Fig. 2:**
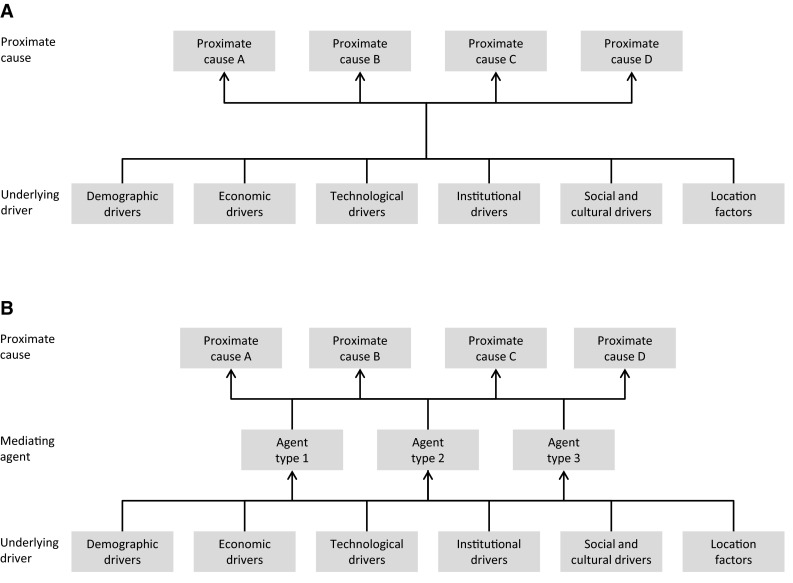
Two conceptually different models for drivers of land use change: **a** Proximate causes and underlying drivers; **b** Explicitly considering agents as moderators between underlying drivers and proximate causes

Many meta-studies of land use change processes are based on the conceptual model of proximate causes and underlying driving forces, as presented by Meyer and Turner (1992) and introduced in meta-studies by Geist and Lambin (2002) (Fig. 2a). Here, proximate causes are the actual process of land use change, such as urbanization, and underlying driving factors are fundamental societal or environmental processes that cause these changes, such as population growth or climate change. Meta-studies benefit from this concept as it facilitates the coding of case studies and wider sample of relevant case studies. Counts of proximate causes and underlying drivers provide a measure of the relative importance of each factor in the case-study population, and the conceptual model allows multifactor causation, which is frequently hypothesized for case studies.

Framing land use change processes in terms of proximate causes and underlying drivers allows for a comparison of meta-study findings across different land use change processes. For instance, both urbanization and agricultural expansion feature prominently as proximate causes in meta-studies of different land use change, including deforestation (Geist and Lambin 2002), dryland degradation (Geist and Lambin 2004) and wetland conversion (van Asselen et al. 2013). Likewise, nearly all meta-studies report conjoint causation: various combinations of biophysical and socioeconomic factors explain observed land use changes (Geist and Lambin 2002, 2004; Keys and McConnell 2005; van Asselen et al. 2013; van Vliet et al. 2015). For example, Robinson et al. (2014) find that land tenure regimes are important drivers of land use change, but that the effects of different regimes depends on the interaction with demographic, economic, and biophysical drivers. While some combinations of drivers are found across different regions, others are region specific. For example, urban expansion is strongly associated with economic growth in China, while it is mainly associated with urban population growth in India and Africa (Seto et al. 2011). Similarly, although market integration contributes to the decline of swidden across the tropics, it is associated with different policies across regions. Policies mainly encourage cattle ranching in South and Central America, while forest conservation and resettlement policies predominate in Southeast Asia (van Vliet et al. 2012). The diversity in land use change processes, reflected in the multiple pathways found in meta-studies, suggests that there is a limit to what extent land use change processes can be generalized.

Proximate causes of land use change often represent the outcomes of human decisions, and underlying drivers constitute the factors which affect these decisions. The motivations, identities, and roles of the different actors and their relationship with each other are frequently not explicitly identified in meta-studies. Consequently, the concept of proximate causes and underlying drivers has been criticized for not explicitly addressing the mediating roles of actors and their decision-making processes (Hersperger et al. 2010). Adding actors as moderators between driving forces and land use changes (Fig. 2b) will improve the potential of meta-studies for understanding decision making in human–environmental systems and also support policy assessment, as many policies are directly addressed at influencing decision making of land use actors (Meyfroidt 2013). For instance, a recent meta-study by (van Vliet et al. 2015) includes actor characteristics as an explicit factor in the coding of case studies, showing that actor characteristics and/or attitudes are moderating factors for different agricultural land use changes observed under otherwise similar conditions.

### Synthesizing complexity in land use science

Many meta-studies implicitly assume a one-directional relationship between land use or land use change and its impacts. This is particularly apparent from meta-studies of effect sizes, i.e., studies that quantitatively assess the impacts of particular land use types or land use changes (see for example Luck 2007 and Gibson et al. 2011). However, this linear perspective does not recognize the complexity of coupled human and natural systems (Liu et al. 2007). In reality, humans adapt their land use to changing environmental conditions as a coping strategy or through environmental cognitions (Meyfroidt 2013), which means that there is a continuous feedback between humans and the environment. Only few meta-studies account for chains of causation and interactions between actors and their environment. Such details are typically lost in the generalization process of coding the case studies. This is especially the case for social, cultural, or economic impacts of land use change, and therefore such impacts are more frequently synthesized in a qualitative way, allowing for a more detailed description of these complex processes (Muchena et al. 2005; DeFries et al. 2007; Cramb et al. 2009). Similar to meta-studies of land use change processes, adding the actors and decision-making processes explicitly in meta-analysis of land use change impacts would enrich our understanding of these impacts and provide valuable information to support policy making as it identifies the role of relevant actors.

The significance of feedbacks in land use change processes and impacts depends on the speed and strength of the responses. The omission of weak feedback mechanisms, such as the feedback between land use change and climate change, can be justifiable because the effect will not be apparent within the time frame of a typical case study. The inclusion of strong feedbacks, on the other hand, such as feedback between actors and their environment, is required to explain the land use change process adequately. The latter is illustrated in the meta-study of Cook et al. (2011), which addresses the interaction among actors and between actors and their environment in residential landscapes. Such feedback can considerably influence land use change processes (Lambin and Meyfroidt 2010) and are, therefore, of prime interest for environmental management and policy. Strict analytic meta-study methods have difficulties capturing feedback and system level responses, and qualitative review methods may be more appropriate for synthesizing complex system descriptions. For example, Moritz et al. (2011) use a qualitative comparative analysis to synthesize risk management strategies in pastoral systems, including their feedback.

Case-study regions are not closed systems. Instead, land use change processes and their impacts are increasingly driven by distant forces such as international trade and transnational land deals (Rudel et al. 2009; Messerli et al. 2013; Meyfroidt et al. 2013). Several meta-studies identify the role of distal drivers, such as global market forces, foreign debts, or trade liberalization (Angelsen and Kaimowitz 1999). However, these meta-studies remain place-based, in that they analyze locations where land use change takes place. The framework that describes such distant forces as “telecoupling” requires researchers to go beyond a place-based perspective and consider flows and processes, such as biomass flows and international trade, which connect sending and receiving regions, including their relevant actors (Liu et al. 2013). The concept of telecoupling has been applied to analyze land use change processes as well as their impacts (Heffernan et al. 2014; Liu 2014; Munroe et al. 2014). Studies of long distance relations have not yet been synthesized in meta-studies in land use science. The conceptual framework of telecoupling provides guidance for the design of meta-studies that analyze the linkages between local changes and the global context. Such perspective is required to advance land use science and address important issues related to global environmental change and food security (Verburg et al. 2013).

### Chains of causation

**Fig. 3:**
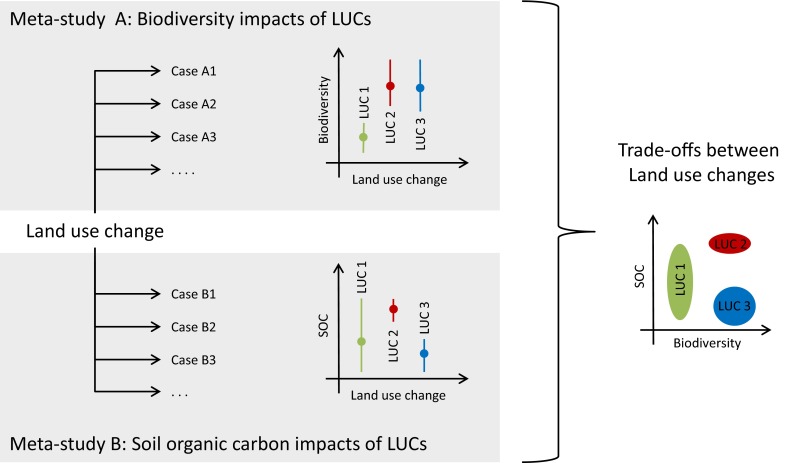
Illustration of the conceptual model of integrating meta-studies to analyze tradeoffs between different land use changes (LUC). Meta-study A analyzes the biodiversity impact of different land use changes, and meta-study B analyzes soil organic carbon (SOC) impacts of different land use changes. The combination of both meta-studies can provide *insights* in the tradeoff between different land use changes; while LSC 2 and 3 yield a higher biodiversity, LUC 1 and LUC 2 generate a higher SOC

Meta-studies reviewed in this paper reveal a strong decoupling of drivers and impacts of land use change. Only few meta-studies assess the link between drivers of land use change, through the changes itself, to their impacts. This is typically a result of their scope and thus their system boundaries. Moreover, including both drivers and impacts of land use changes in a single meta-analysis comes at risk of becoming overly complex, and the number of relevant case studies reporting on the link between drivers and impacts may become prohibitively small. Archetypical combinations of driving factors leading to typical land use changes and associated impacts are, therefore, not identified. On the other hand, understanding these links is important, especially in order to design effective policies to mitigate undesired land use change impacts. One way to reconcile the complexity of land use dynamics with the inherent necessity to simplify cases in meta-studies is to design case studies comparison to combine a common reference framework, in order to enhance comparability across cases, with narrative information to also draw on the rich qualitative background of each case (Persha et al. 2011; Meyfroidt et al. 2014). Although this approach may not allow to draw generalizable conclusions about the outcomes of different drivers or land use processes, it may provide general insights on the chains of causal mechanisms underlying land changes. Alternatively, an integrative analysis of existing meta-studies could track the chain of causation from drivers through land use change to consequences by combining multiple meta-studies (Fig. 3).

Meta-studies that implement the conceptual framework of proximate causes and underlying drivers often relate different land use change processes through proximate causes. For example, expansion of agricultural land was found as a proximate cause for wetland conversion (van Asselen et al. 2013), and urban expansion was found as a proximate cause of the contraction of agricultural land in Europe (van Vliet et al. 2015). This indicates that meta-study findings framed as underlying drivers and proximate causes can be combined to relate changes in different land use types, potentially informing a more integrated theory of land use change. Besides theory development, such analysis would be very beneficial for the development of land use models that aim to simulate multiple types of land use change. Such models are frequently used for scenarios studies and policy assessments (van Delden et al. 2010; Sohl and Claggett 2013). Meta-studies can greatly enhance the scientific basis of these models by informing their design and implementation, and thereby their acceptability for policy applications (Magliocca et al. 2015a).

**Fig. 4:**
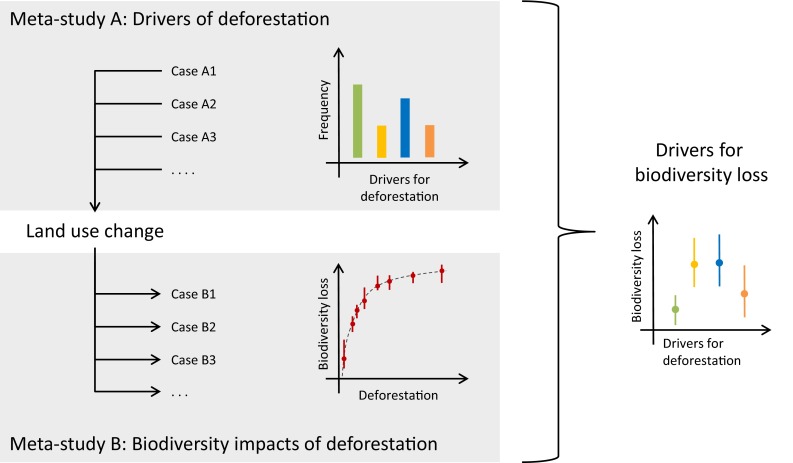
Illustration of a conceptual model for integrating meta-studies of drivers and meta-studies of impacts of land use change. Meta-study A characterizes the frequency with which drivers underlie deforestation and meta-study B characterizes biodiversity loss as a function of deforestation. Combining both findings will inform how specific drivers of land use change contribute to biodiversity loss

Meta-studies of impacts of land use change typically focus on single impacts, or a group of related impacts, such as the species abundance for several taxa or fluxes of several nutrients. However, land use changes often result in multiple different impacts, with possibly positive effects on one dimension and negative on others. For example, Marczak et al. (2010) conclude that the response of terrestrial species to riparian buffers was not consistent between taxonomic groups, and Tscharntke et al. (2011) assess a wider range of consequences of the removal of shade trees in agroforestry systems, including biodiversity, agricultural production, and pollination. Although case studies investigating such tradeoffs are becoming more common (Raudsepp-Hearne et al. 2010; Willemen et al. 2010; Phalan et al. 2011), only few meta-studies assess multiple impacts of land use changes. Combining studies on the impacts of land use changes could allow for an analysis of tradeoffs and synergies between multiple different impacts, allowing for more comprehensive assessments of land use change impacts (Fig. 4). Explicitly addressing tradeoffs and synergies provides information to make more balanced policies, accounting for multiple impacts, rather than focused on one impact only.

## Conclusions

Empirical, place-based, research in case studies is a central component of land use science, but provides limited opportunities for generalization, because results are inherently context dependent. Meta-studies allow to go beyond single cases and provide more comprehensive results, for example to develop theories, parameterize models, or to support policy design. This review has discussed both meta-studies addressing land use change processes, and meta-studies addressing the impacts of land use changes. The latter types primarily assess land use change impacts on biodiversity metrics and biogeochemical indicators, while less attention is paid to hydrologic impacts, food production, and especially to socioeconomic impacts. The majority of meta-studies focus on forest or agricultural land uses, while urban areas, wetlands, and grasslands have received much less attention.

The breadth of case-study evidence in land use science requires meta-studies to simplify the applied conceptualization of land use change processes and their impacts in the meta-study design in order to make findings comparable. As a consequence, meta-studies have been very successful in identifying direct cause–effect relations, but not in analyzing more complex chains of causation and feedback mechanisms. Standardizing cases’ experimental design has been proposed as a way to increase comparability of empirical results (Turner et al. 1994; Carpenter et al. 2012). Guidelines may improve the comparability of case studies, but a completely standardized procedure will be neither feasible nor desirable in many cases, since case studies are conducted with different objectives and innovation is required to uncover new insights.

To further improve our understanding of land use change processes and their impacts, we identified opportunities for more integrated analysis of land use change processes and their impacts using meta-studies. These opportunities relate to the design of meta-studies, the combination of meta-study results, and the application of meta-study results. First, future meta-studies could address the role of actors and decision making in land use changes explicitly, as they moderate the effect from drivers to land use changes. Then, while meta-studies typically focus on either land use change processes or land use change impacts, combining meta-studies would allow analyzing the relation between land use change drivers through land use changes to their impacts. Similarly, combining meta-studies of land use change impacts allows analyzing tradeoffs between different impacts of the same land use change. Also, combining meta-studies of different land use change processes would support building theory of land use change that relates different land use change processes with each other and with their underlying drivers.

## Electronic supplementary material

Supplementary material 1 (PDF 708 kb)
