# Supplementary material for: Meta-studies in land use science: Current coverage and prospects
Source: Ambio. 2015 Sep 25;45(1):15–28. doi: 10.1007/s13280-015-0699-8 (PMC4709351; doi:10.1007/s13280-015-0699-8)
Supplement: Supplementary file 1 — Supplementary material 1 (PDF 708 kb) [file 13280_2015_699_MOESM1_ESM.pdf]

AMBIO

Electronic Supplementary Material
This supplementary material has not been peer reviewed

Title: Meta-studies in land use science: Current status and prospects

Authors: Jasper van Vliet, Nick Magliocca, Bianka Buchner, Elizabeth Cook, José M. Rey Benayas, Erle C. Ellis, Andreas Heinimann, Eric Keys, Tien Ming Lee, Jianguo Liu, Ole Mertz, Patrick Meyfroidt, Mark Moritz, Christopher Poeplau, Brian E. Robinson, Ralf Seppelt, Karen C. Seto, Peter H. Verburg

Systematic review protocol

We searched ISI web of Knowledge for all meta-studies that reported:

- Human modifications and appropriations of natural processes as causes of observed land cover change, or
- How the impacts of land-use change directly affect natural and/or human system outcomes, or
- A combination of these two

A target set of 12 known meta-studies was first identified to represent the expected range of synthesis methods and research topics present in LCS (Angelsen and Kaimowitz 1999; Guo and Gifford 2002; Geist and Lambin 2004; Keys and McConnell 2005; Misselhorn 2005; Pagdee et al. 2006; Scanlon et al. 2006; Tonitto et al. 2006; Rudel 2007; Schueler et al. 2009; Seto et al. 2011; van Vliet et al. 2012). Keywords are selected from these meta-studies such that all target meta-studies appear in the search results. This yielded the following search string:

TS=(((meta AND (study OR analy*)) OR "case studies" OR synthes* OR intergrat* OR (meta- AND (study OR anal*)) OR (geographic* AND database)) AND ((land AND (use OR cover)) OR (land- AND (use OR cover)) OR "food *security" OR ((environmental OR forest OR land OR resource* OR "common pool" OR (agricultur* AND production)) AND (management OR economics OR cultivat*))) AND (((proximate OR direct OR indirect) AND causes) OR "driv*" OR crop* OR urban OR rural OR deforestation OR agricultur* OR forest OR dryland OR desertification OR expansion OR intensification OR conservation))

Refined by: Web of Science Categories=(ECOLOGY OR ENVIRONMENTAL SCIENCES OR ENVIRONMENTAL STUDIES OR WATER RESOURCES OR PLANT SCIENCES OR BIODIVERSITY CONSERVATION OR AGRICULTURE MULTIDISCIPLINARY OR GEOGRAPHY OR SOIL SCIENCE OR METEOROLOGY ATMOSPHERIC SCIENCES OR PLANNING DEVELOPMENT OR AGRICULTURAL ECONOMICS POLICY OR ECONOMICS OR ENGINEERING CIVIL OR SOCIOLOGY OR MULTIDISCIPLINARY SCIENCES OR BIOLOGY)

We further narrowed the number of studies included by the additional condition that:

- Meta-studies were defined as studies that are secondary studies, i.e. based on other previously published primary research, and that are systematic in their analysis, i.e. excluding literature reviews in which the translation between cases and the meta-study findings was not tractable.
- Studies should report on a comparison of two or more discrete situations either in time (before/after) or in space (i.e. using space time substitution).
- Studies should analyze land use change processes or their impacts at landscape scale. As a consequence studies that assess individual crops or crop management practices are not included, but studies that assess farm systems (such as organic versus conventional) are included.
- Studies must be generalizable beyond the set of sample cases analyzed to describe large scale trends
- Be representative of a globally or regionally relevant process, and
- Not be constrained by political boundaries alone.

The systematic search was originally performed in 2012 (Magliocca et al. 2015), and subsequently updated to include all studies published in or before 2014. In addition, we added three studies that did not appear in our search results but were identified by workshop participants, and we added another three studies that did not appear in our search results but were identified by the reviewers.

Overview of meta-studies in land use science

Table S1: Meta-studies analyzing land use change processes.

Meta-study	Land change process explained	Regional coverage	Studies	Observations	Synthesis method*
**Agriculture**					
Baumgart-Getz et al. (2012)	Adoption of agricultural best management practices	USA	46	46	COMA
Current et al. (1995)	Adoption of agroforestry by farmers	Latin America	21	21	CSC
Keys and McConnell (2005)	Intensification and expansion of agricultural land	Tropics	91	108	CSC
Meyfroidt et al. 2014)	Commodity crop expansion	Tropics	6	6	VOMA
Rey Benayas et al. (2007)	Abandonment of agricultural land	Global	45	45	COMA
Rindfuss et al. (2007)	Agricultural expansion in forest frontiers	Tropics	7	7	CSC
Schmidt-Vogt et al. 2009)	Changes in fallow length and extent of swidden, conversion of swidden	SE Asia	67	151	VOMA
van Vliet et al. (2012)	Changes in fallow length and extent of swidden, conversion of swidden	Tropics	111	157	MAES
van Vliet et al. (2013)	Changes in cropland area	Sahel	25	50	VOMA
van Vliet et al. (2015)	Intensification and disintensification of agricultural land use	Europe	137	218	COMA
**Forests**					
Angelsen and Kaimowitz (1999)	Tropical deforestation	Tropics	146	146	CSMDA
Geist and Lambin (2002)	Tropical deforestation	Tropics	95	152	VOMA
Mondal and Nagendra (2011)	Deforestation and forest degradation in tiger landscapes	Asia	27	24	VOMA
Pagdee et al. (2006)	Protection of community forests	Global	31	69	VOMA
Robinson et al. (2014)	Influence of land tenure form and security on deforestation and forest degradation	Tropics	36	118	CSMDA
Rudel (2007)	Agents for tropical deforestation (from state run to enterprise led deforestation)	Tropics	268	268	COMA
Rudel et al. (2009)	Tropical deforestation and regrowth	Tropics	268	268	COMA
Rutte (2011)	Sacred areas as community based conservation of natural areas	Global	18	21	VOMA
**Urban**					
Seto et al. (2011)	Expansion of urban areas	Global	181	326	CSMDA
**Wetland**					
van Asselen et al. (2013)	Conversion of wetlands into other land uses	Global	88	105	COMA
**Multiple land uses**					
Geist and Lambin (2004)	Dryland degradation and desertification	Global	54	132	VOMA
Munteanu et al. (2014)	Increase and decrease of forest and agricultural land area	Eastern Europe	66	102	VOMA

Table S2: Meta-studies analyzing the impacts of different land uses or land use change processes on biodiversity.

Meta-study	Land uses or land use change processes included	Specific impact	Regional coverage	Studies	Observations	Synthesis method*
**Agriculture**						
Attwood et al. (2008)	Intensification of agricultural land use	Arthropod species richness and abundance	Global	259	259	MAES
Batáry et al. (2011)	Difference between simple and complex landscapes under agri-environmental management	Species richness and abundance of plants and animals	Global	93	223	MAES
Bengtsson et al. (2005)	Organic farming and conventional farming	Species richness in various taxa	Global	63	180	VOMA
Bremer and Farley (2010)	Conversion to plantations for wood production, soil and water conservation, and carbon sequestration	Plant species richness	Global	36	126	CSMDA
Chaplin-Kramer et al., 2011)	Different levels of landscape complexity	Pests, natural enemies and/or pest control	Global	46	159	MAES
De Beenhouwer et al. (2013)	Conversion of forests into cacao and coffee agroforestry systems	Decline in biodiversity and other ecosystem services	Tropics	74	202	MAES
Fletcher et al. (2011)	Conversion to biofuel cropping	Vertebrate diversity and vertebrate abundance	USA	15	272	MAES
Garratt et al. (2011)	Organic and conventional agriculture	Arthropod biodiversity	Global	71	71	COMA
Kleijn and Sutherland (2003)	Adoption of agri-environmental schemes	General species abundance	Europe	62	62	CSC
Letourneau et al. (2011)	Difference in monoculture cropping systems and plant diversity	Herbivore reduction and herbivore enemy increase	Global	45	552	MAES
Philpott et al. (2008)	Difference in management of coffee plantations	Species richness of ants, birds and trees	Tropics	27	207	MAES
Queiroz et al. (2014)	Farmland abandonment	General biodiversity	Global	276	276	VOMA
Rahmann (2011)	Organic and conventional farming systems	General biodiversity	Global	396	396	VOMA
Ramírez and Simonetti (2011)	Structure and density of commercial plantations	Mammal diversity (size and dietary group)	Global	45	81	CSMDA
Tuck et al. (2014)	Organic and conventional farming, and land use intensity	Species richness for various groups of species	Global	94	184	MAES
**Forests**						
Bhagwat and Rutte (2006)	Sites protected for sacred reasons	Biodiversity conservation	Global	98	98	VOMA
Bogdziewicz and Zwolak (2014)	Clear-cutting of temperate and boreal forests	Small mammal abundance	Europe	20	156	MAES
Boucher et al. (2011)	Old-growth forest loss and fragmentation due to logging	Biodiversity loss due to edge effects	North-America	9	9	CSC

Cristescu et al. (2012)	Restoration after mining	Fauna recolonization	Australia	71	849	VOMA
De Frenne et al. (2011)	Ancient forests and post-agriculture forests	Recovery rate of floristic species	Europe	18	812	MAES
Duguid and Ashton (2013)	Forest management and regeneration treatment	Plant species richness	North America	37	96	MAES
Gibson et al. (2011)	Forest disturbance and forest conversion	Biodiversity for a wide range of taxa	Tropics	138	2220	MAES
Gray et al. (2007)	Undisturbed forests and selective logging	body size, local population size, and geographic range size of different feeding guilds of birds	Tropics	57	2354	CSMDA
Hill and Hamer (2004)	Undisturbed forests, selected logging and shifting cultivation	Biodiversity of birds	Tropics	37	37	CSMDA
Holloway and Smith (2011)	Various harvesting practices	Abundance of northern flying squirrels	North America	14	14	MAES
Karthik et al. (2009)	Forest recovery following shifting cultivation	Biomass accumulation and species richness	Global	43	43	CSC
Koh (2007)	Deforestation and habitat loss	Butterfly species richness	SE Asia	20	20	CSC
Marczak et al. (2010)	Riparian buffers and unharvested riparian sites	Species abundance of various taxa	Global	27	519	MAES
Mori and Kitagawa (2014)	Selective logging and retention logging	General species diversity	Global	50	221	MAES
Paillet et al. (2010)	Forest structure and composition in managed and unmanaged forests	General species richness	Europe	49	120	MAES
Putz et al. (2012)	Forest harvesting	Capacity to sustain timber production, retain species, and conserve carbon stocks	Tropics	109	109	CSMDA
Rey Benayas et al. (2009)	Forest restoration	Biodiversity and provisioning of ecosystems services	Global	89	526	MAES
Schlossberg and King (2009)	Forest logging	Bird population's response after logging	North America	11	11	MAES
Schmidt et al. (2011)	Harvesting non-timber forest products	Population responses to harvest	Global	48	134	MAES
Sodhi et al. (2009)	Deforestation and forest degradation	Ecological health: richness, abundance, and demographics	SE Asia	120	1074	MAES
Ticktin (2004)	Harvesting non-timber forest products	Ecological consequences of harvesting non timber forest products	Global	70	70	CSC
Vanderwel et al. (2007)	Forest harvesting	Bird species responses	North America	42	435	MAES
Vellend et al. (2007)	Old forest and young forests	Forest species diversity	North America and Europe	11	1446	CSDA
**Urban**						
Colding and Folke	Golf courses and other green area habitats	Species richness and preservation of fauna	Global	17	190	CSMDA

(2008)		of conservation concern				
Cook et al. (2011)	Various types of garden management	Wide range of ecological properties and functions	North America	256	256	VOMA
Hahs et al. (2009)	Effect of urban developments	Plant extinction rates	Global	22	22	CSMDA
Luck (2007)	Human population densities	Species richness for various taxa	Global	85	401	MAES
Martinson and Raupp (2013)	Urban areas (compared to rural forested sites)	Species richness and abundance of beetles	Global	18	658	MAES
Stewart et al. (2007)	Absence and presence of wind farms, and the time since their development	Bird collision with turbines, habitat loss and disturbance	Global	15	19	MAES
**Multiple land uses**						
Barral et al. (2015)	Restauration of agroecosystems: land sharing versus land sparing	Biodiversity, as well as a number of other ecosystem services	Global	54	154	MAES
Felton et al. (2010)	Pastures and timber plantations	species richness and abundance for plants, invertebrates, reptiles/amphibians, mammals, and birds	Global	36	145	MAES
Flynn et al. (2009)	Natural and agricultural land use	Species richness and diversity of plant, bird and mammal communities	Global	20	1670	MAES
Gerstner et al. (2014)	Multiple land use types	Species richness of plants	Global	375	572	MAES
Hadley and Betts (2012)	Habitat loss and habitat fragmentation	Animal mediated pollination	Global	303	303	VOMA
Kennedy et al. (2013)	Landscape composition and configuration, farm management	Abundance and species richness of bee pollinators	Global	39	605	MAES
Laliberté et al. (2010)	Various land use types along an intensity gradient	Functional redundancy and response diversity of plant communities	Global	18	18	MAES
Mantyka-pringle et al. (2012)	Habitat loss in combination with climate change	Species density and diversity of a wide range of taxa	Global	168	1779	MAES
Montero-Castaño and Vilà (2012)	Landscape and habitat alteration	Impact on pollinators and the pollination services	Global	58	143	MAES
Murphy and Romanuk (2014)	Disturbed and undisturbed habitats	Decline in species richness	Global	245	327	COMA
Nichols et al. (2007)	Disturbance gradient from selectively logged forest to annual crops, pastures and clear-cuts	Beetle abundance and species richness	Global	33	33	MAES
Prieto-Benítez and Méndez (2011)	Various land management types, including insecticides, forest fragmentation and logging, and farmland abandonment.	Spider abundance and spider biodiversity	Global	174	197	MAES
Tylianakis et al. (2008)	Habitat loss, fragmentation, and increased land use intensity	Pollination, species richness, species interaction	Global	688	688	VOMA
Winfree et al. (2009)	Habitat loss	Pollination, species richness	Global	54	130	MAES
Winfree et al. (2011)	Habitat loss	Pollination, species richness	Global	265	674	MAES

Table S3: Meta-studies analyzing the impacts of different land uses or land use change processes on biogeochemical processes. See main text for explanations of the synthesis methods.

Meta-study	Land uses or land use change processes included	Specific impact	Regional coverage	Studies	Observations	Synthesis method*
**Agriculture**						
Aguilera et al. (2013)	Recommended management practices of Mediterranean cropland	Changes in soil organic carbon and carbon sequestration	Mediterranean regions	79	174	MAES
Govaerts et al. (2009)	Conventional agricultural and conservation agriculture	Carbon sequestration and soil organic carbon	Global	40	127	CSC
Huang et al. (2011)	Various rice cropping systems	Soil organic carbon stocks in paddy fields	China	44	44	MAES
Jarecki and Lal (2003)	Recommended management practices for cropland	Soil organic matter concentration	Global	41	430	VOMA
MacDonald et al. (2012)	Abandoned and remaining agricultural areas	Changes in soil phosphorous	Global	94	94	MAES
Ogle et al. (2005)	Long-term cultivation, setting-aside land, changing tillage management, and varying cropping practices	Change in soil organic carbon	Global	126	126	MAES
Ogle et al. (2012)	Tillage or no-tillage in land management	Change in soil carbon stock and crop productivity	North America	74	1040	MAES
Skinner et al. (2014)	Organic and non-organic agricultural management	Greenhouse gas fluxes	Global	19	25	VOMA
Tonitto et al. (2006)	Conventional inorganic fertilization and diversified land management	Yields, nitrate leaching, and soil nitrates	North America	31	635	MAES
Tuomisto et al. (2012)	Organic farming and conventional farming	Soil organic matter, nutrient losses, eutrophication per unit area and per unit product	Europe	71	257	MAES
**Forests**						
Bárcena et al. (2014)	Afforestation	Soil organic carbon changes	Northern Europe	18	119	MAES
Berthrong et al. (2009)	Afforestation	Mineral soil quality, including pH, sodium, exchangeable cations, organic carbon, and nitrogen	Global	71	153	MAES
Bonner et al. (2013)	Secondary forests and plantations	Aboveground biomass accumulation	Tropics	48	330	MAES
Cook et al. (2011)	Garden management (fertilization, mowing, irrigation)	Wide range of ecological properties and functions	North America	256	256	VOMA
Hergoualc'h and Verchot (2012)	Deforestation of peat swamp forests	Changes in peat methane fluxes	SE Asia	6	16	CSC
Hoogmoed et al. (2012)	Afforestation of pastures	Soil carbon, soil nitrogen and C:N ratio	Australia	7	25	VOMA
Kauffman et al.	Deforestation and secondary forest	Carbon pools and carbon accumulation	Latin America	7	13	CSC

(2009)	establishment					
Lawrence et al. (2010)	Biomass recovery in shifting cultivation cycles	Biomass recovery after cultivation	Global	6	31	CSC
Li et al. (2012)	Afforestation of cropland and pastures	Dynamics of soil C and N stocks	Global	70	292	CSMDA
Liao et al. (2012)	Plantation forests and natural forests	Soil properties and nutrients	Global	73	560	MAES
Marín-Spiotta and Sharma (2013)	Plantation, secondary and reference forests	Soil carbon	Tropics	81	510	MAES
Putz et al. (2012)	Forest harvesting	Capacity to sustain timber production, retain species, and conserve carbon stocks	Tropics	109	109	CSMDA
Shi et al. (2013)	Afforestation of grasslands and croplands	Deep soil organic carbon	Global	56	63	MAES
van Kooten et al. (2004)	Forest conservation, tree planting and agroforestry	Costs for carbon sequestration	Global	55	981	CSMDA
Zhou et al. (2013)	Partial cutting	Carbon in aboveground biomass, understory, forest floor and soil	Global	81	748	MAES
**Multiple land uses**						
Don et al. (2011)	Conversion of forests and afforestation	Soil organic carbon stocks	Tropics	153	385	MAES
Guo and Gifford (2002)	Various land use changes	Soil carbon stocks	Global	74	537	MAES
Kim et al. (2013)	Agricultural and natural lands	Background nitrous oxide emissions	Global	362	907	MAES
Lu et al. (2008)	Various land cover types	Red soil rehabilitation	China	55	128	MAES
Poeplau et al. (2011)	Several different land use change types	Soil organic carbon dynamics	Global	95	322	MAES
Powers et al. (2011)	Several different land use change types	Change in soil carbon stock	Tropics	80	837	MAES
Wright and Fridley (2010)	Old field succession	Biomass accumulation	North America	30	30	CSMDA
Ziegler et al. (2012)	Change from swidden to other land use	Carbon emission and sequestration	SE Asia	250	250	CSC

Table S4: Meta-studies analyzing the impacts of different land uses or land use change processes on hydrology or water quality.

Meta-study	Land uses or land use change processes included	Specific impact	Regional coverage	Studies	Observations	Synthesis method*
**Agriculture**						
Ilstedt et al. (2007)	Afforestation and tree planting in agriculture	Water infiltration	Tropics	4	14	MAES
Keatley et al. (2011)	Expansion and intensification of agricultural land	Freshwater eutrophication	Global	67	67	CSMDA
Taranu and Gregory-Eaves	Proportion of agricultural land in a catchment	Lake trophic state	Global	12	358	CSC

(2008)						
**Forests**						
Farley et al. (2005)	Afforestation, original vegetation type, plantation species, and plantation age	Annual runoff and low flow	Global	20	504	CSDA
Richardson and Béraud (2014)	Logging of riparian forest sites	Water chemistry, algae, organic particles in the water, benthic invertebrates and aquatic insects	Global	18	96	MAES
Salemi et al. (2012)	(Removal of) riparian forests	Water yield and variation	Global	7	10	VOMA
**Urban**						
Schueler et al. (2009)	Proportion of impervious surface in a watershed	Water quality, biological diversity, geomorphology, hydrology	North America	65	65	COMA
**Multiple land uses**						
Das et al. (2009)	Land use composition in a watershed	Concentrations of Fe, Mn, Cu, dissolved organic matter	Global	30	135	MAES
Kim and Jackson (2012)	Distribution of main land use types and vegetation cover	Groundwater recharge response	Global	206	600	CSC
Maetens et al. (2012)	Wide range of land cover types, including construction, bare, cropland, grassland and (semi-) natural	Annual runoff, annual runoff coefficient and annual soil loss	Europe and Mediterranean	10	227	CSMDA
Petheram et al. (2002)	Vegetation cover (annual, perennial, trees) in a watershed	Groundwater recharge response	Australia	41	41	VOMA
Scanlon et al. (2006)	Climate variability and vegetation cover change	Groundwater recharge response	Global	140	140	CSC
Smucker and Detenbeck (2014)	Out-of-stream restauration practices, including wetland creation, riparian vegetation and stormwater ponds	Various ecosystem attributes, including	Global	38	319	MAES
Srinivasan et al. (2012)	Agricultural and urban water demand,	Various effects of water quantity on human wellbeing	Global	22	22	COMA
Wickham et al. (2008)	Land use distribution in a watershed	Watershed nutrient yield	North America	167 (sites)	1228	CSDA

Table S5: Meta-studies analyzing the impacts of different land uses or land use change processes on food production.

Meta-study	Land uses or land use change processes included	Specific impact	Regional coverage	Studies	Observations	Synthesis method*
**Agriculture**						
Brouder and Gomez-Macpherson	Conventional and conservation smallholder agriculture	Crop yield for maize, rice, cowpea and sorghum	Subsahara Africa and South Asia	53	56	VOMA

(2014)						
Iverson et al. (2014)	Different crop rotation schemes	Trade-off between crop yield and biodiversity	Global	26	301	VOMA
Ponisio et al. (2014)	Conventional and organic agriculture	Crop yields for various crop types	Global	115	1071	MAES
De Ponti et al. (2012)	Conventional and organic agriculture	Crop yields for various crop types	Global	135	362	VOMA
Seufert et al. (2012)	Conventional and organic agriculture	Crop yields for various crop types	Global	62	316	MAES
Rivest et al. (2013)	Scattered trees on pastures	Pasture yield	Global	27	73	MAES
Rusinamhodzi et al. (2011)	Conventional and conservation agriculture	Maize grain yields	Southern Africa	26	26	VOMA

Table S6: Meta-studies analyzing the impacts of different land uses or land use change processes on livelyhoods and other socio-economic factors.

**Meta-study**	**Land uses or land use change processes included**	**Specific impact**	**Regional coverage**	**Studies**	**Observations**	**Synthesis method***
**Agriculture**						
Bennett and Franzel (2013)	Organic and resource conserving, and conventional agriculture	Livelihoods: yields, food security, income	Tropics	31	31	COMA
van Vliet et al. (2013)	Changes in cropland area	Environmental and livelihood impacts of cropland changes	Sahel	25	50	VOMA
**Grassland**						
Muchena et al. (2005)	Land degradation	Consequences for food security and rural livelihoods	Africa	14	21	CSC
**Multiple land uses**						
Cramb et al. (2009)	Intensification and extensification of swidden	Economic and cultural consequences of changes in swidden	SE Asia	6	6	CSC

* Synthesis methods: cross-site comparisons (CSC) are syntheses of unsystematically selected cases studies; cross-site data analyses (CSDA) are statistical analyses identifying patterns across aggregate variable data; cross-site meta-data analyses (CSMDA) and meta-analyses of effect sizes (MAES) are statistical analyses (e.g. regression) across data values and the magnitudes of effects reported in systematically selected case studies respectively; variable-oriented meta-analyses (VOMA) are statistical analyses identifying links between coded variables across cases; and case-oriented meta-analyses (COMA) are analyses of the relationships between coded variables within and across cases. For a more elaborate description of these methods, see (Magliocca et al. 2015)

References

Aguilera, E., L. Lassaletta, A. Gattinger, and B. S. Gimeno. 2013. Managing soil carbon for climate change mitigation and adaptation in Mediterranean cropping systems: A meta-analysis. *Agriculture, Ecosystems and Environment* 168: 25–36. doi:10.1016/j.agee.2013.02.003.

Angelsen, A., and D. Kaimowitz. 1999. Rethinking the causes of deforestation: lessons from economic models. *The World Bank Research Observer* 14: 73–98. doi:10.1093/wbro/14.1.73.

Van Asselen, S., P. H. Verburg, J. E. Vermaat, and J. H. Janse. 2013. Drivers of wetland conversion: a global meta-analysis. *PloS one* 8: e81292. doi:10.1371/journal.pone.0081292.

Attwood, S. J., M. Maron, A. P. N. House, and C. Zammit. 2008. Do arthropod assemblages display globally consistent responses to intensified agricultural land use and management? *Global Ecology and Biogeography* 17: 585–599. doi:10.1111/j.1466-8238.2008.00399.x.

Bárcena, T. G., L. P. Kiær, L. Vesterdal, H. M. Stefánsdóttir, P. Gundersen, and B. D. Sigurdsson. 2014. Soil carbon stock change following afforestation in Northern Europe: A meta-analysis. *Global Change Biology*. doi:10.1111/gcb.12576.

Barral, M. P., J. M. Rey Benayas, P. Meli, and N. O. Maceira. 2015. Quantifying the impacts of ecological restoration on biodiversity and ecosystem services in agroecosystems: A global meta-analysis. *Agriculture, Ecosystems & Environment* 202: 223–231. doi:10.1016/j.agee.2015.01.009.

Batáry, P., A. Báldi, D. Kleijn, and T. Tscharntke. 2011. Landscape-moderated biodiversity effects of agri-environmental management: a meta-analysis. *Proceedings. Biological sciences / The Royal Society* 278: 1894–902. doi:10.1098/rspb.2010.1923.

Baumgart-Getz, A., L. S. Prokopy, and K. Floress. 2012. Why farmers adopt best management practice in the United States: a meta-analysis of the adoption literature. *Journal of environmental management* 96: 17–25. doi:10.1016/j.jenvman.2011.10.006.

De Beenhouwer, M., R. Aerts, and O. Honnay. 2013. A global meta-analysis of the biodiversity and ecosystem service benefits of coffee and cacao agroforestry. *Agriculture, Ecosystems & Environment* 175: 1–7. doi:10.1016/j.agee.2013.05.003.

Bengtsson, J., J. Ahnström, and A. C. Weibull. 2005. The effects of organic agriculture on biodiversity and abundance: A meta-analysis. *Journal of Applied Ecology* 42: 261–269. doi:10.1111/j.1365-2664.2005.01005.x.

Bennett, M., and S. Franzel. 2013. Can organic and resource-conserving agriculture improve livelihoods? A synthesis. *International Journal of Agricultural Sustainability* 11: 193–215. doi:10.1080/14735903.2012.724925.

Berthrong, S. T., E. G. Jobbágy, and R. B. Jackson. 2009. A global meta-analysis of soil exchangeable cations, pH, carbon, and nitrogen with afforestation. *Ecological Applications* 19: 2228–2241. doi:10.1890/08-1730.1.

Bhagwat, S. A., and C. Rutte. 2006. Sacred groves: potential for biodiversity management. *Frontiers in Ecology and the Environment* 4: 519–524. doi:10.1890/1540-9295(2006)4[519:SGPFBM]2.0.CO;2.

Bogdziewicz, M., and R. Zwolak. 2014. Responses of small mammals to clear-cutting in temperate and boreal forests of Europe: A meta-analysis and review. *European Journal of Forest Research*. doi:10.1007/s10342-013-0726-x.

Bonner, M. T. L., S. Schmidt, and L. P. Shoo. 2013. A meta-analytical global comparison of aboveground biomass accumulation between tropical secondary forests and monoculture plantations. *Forest Ecology and Management* 291: 73–86. doi:10.1016/j.foreco.2012.11.024.

Boucher, Y., M.-H. St-Laurent, and P. Grondin. 2011. Logging-induced edge and configuration of old-growth forest remnants in the eastern North American boreal forests. *Natural Areas Journal* 31: 300–306. doi:10.3375/043.031.0313.

Bremer, L. L., and K. A. Farley. 2010. Does plantation forestry restore biodiversity or create green deserts? A synthesis of the effects of land-use transitions on plant species richness. *Biodiversity and Conservation* 19: 3893–3915. doi:10.1007/s10531-010-9936-4.

Brouder, S. M., and H. Gomez-Macpherson. 2014. The impact of conservation agriculture on smallholder agricultural yields: A scoping review of the evidence. *Agriculture, Ecosystems and Environment*. doi:10.1016/j.agee.2013.08.010.

Chaplin-Kramer, R., M. E. O'Rourke, E. J. Blitzer, and C. Kremen. 2011. A meta-analysis of crop pest and natural enemy response to landscape complexity. *Ecology letters* 14: 922–32. doi:10.1111/j.1461-0248.2011.01642.x.

Colding, J., and C. Folke. 2008. The role of golf courses in biodiversity conservation and ecosystem management. *Ecosystems* 12: 191–206. doi:10.1007/s10021-008-9217-1.

Cook, E. M., S. J. Hall, and K. L. Larson. 2011. Residential landscapes as social-ecological systems: a synthesis of multi-scalar interactions between people and their home environment. *Urban Ecosystems* 15: 19–52. doi:10.1007/s11252-011-0197-0.

Cramb, R. A., C. J. P. Colfer, W. Dressler, P. Laungaramsri, Q. T. Le, E. Mulyoutami, N. L. Peluso, and R. L. Wadley. 2009. Swidden transformations and rural livelihoods in Southeast Asia. *Human Ecology* 37: 323–346. doi:10.1007/s10745-009-9241-6.

Cristescu, R. H., C. Frère, and P. B. Banks. 2012. A review of fauna in mine rehabilitation in Australia: Current state and future directions. *Biological Conservation* 149: 60–72. doi:10.1016/j.biocon.2012.02.003.

Current, D., E. Lutz, and S. J. Scherr. 1995. The costs and benefits of agroforestry to farmers. *The World Bank Research Observer* 10: 151–180. doi:10.1093/wbro/10.2.151.

Das, B., R. Nordin, and A. Mazumder. 2009. Watershed land use as a determinant of metal concentrations in freshwater systems. *Environmental Geochemistry and Health*. doi:10.1007/s10653-008-9244-z.

Don, A., J. Schumacher, and A. Freibauer. 2011. Impact of tropical land-use change on soil organic carbon stocks - a meta-analysis. *Global Change Biology* 17: 1658–1670. doi:10.1111/j.1365-2486.2010.02336.x.

Duguid, M. C., and M. S. Ashton. 2013. A meta-analysis of the effect of forest management for timber on understory plant species diversity in temperate forests. *Forest Ecology and Management*. doi:10.1016/j.foreco.2013.04.009.

Farley, K. A., E. G. Jobbagy, and R. B. Jackson. 2005. Effects of afforestation on water yield: a global synthesis with implications for policy. *Global Change Biology* 11: 1565–1576. doi:10.1111/j.1365-2486.2005.01011.x.

Felton, A., E. Knight, J. Wood, C. Zammit, and D. Lindenmayer. 2010. A meta-analysis of fauna and flora species richness and abundance in plantations and pasture lands. *Biological Conservation* 143: 545–554. doi:10.1016/j.biocon.2009.11.030.

Fletcher, R. J., B. A. Robertson, J. Evans, P. J. Doran, J. R. Alavalapati, and D. W. Schemske. 2011. Biodiversity conservation in the era of biofuels: risks and opportunities. *Frontiers in Ecology and the Environment* 9: 161–168. doi:10.1890/090091.

Flynn, D. F. B., M. Gogol-Prokurat, T. Nogeire, N. Molinari, B. T. Richers, B. B. Lin, N. Simpson, M. M. Mayfield, et al. 2009. Loss of functional diversity under land use intensification across multiple taxa. *Ecology letters* 12: 22–33. doi:10.1111/j.1461-0248.2008.01255.x.

De Frenne, P., L. Baeten, B. J. Graae, J. Brunet, M. Wulf, A. Orczewska, A. Kolb, I. Jansen, et al. 2011. Interregional variation in the floristic recovery of post-agricultural forests. *Journal of Ecology* 99: 600–609. doi:10.1111/j.1365-2745.2010.01768.x.

Garratt, M. P. D., D. J. Wright, and S. R. Leather. 2011. The effects of farming system and fertilisers on pests and natural enemies: A synthesis of current research. *Agriculture, Ecosystems and Environment*. doi:10.1016/j.agee.2011.03.014.

Geist, H. J., and E. F. Lambin. 2002. Proximate causes and underlying driving forces of tropical deforestation. *BioScience* 52: 143. doi:10.1641/0006-3568(2002)052[0143:PCAUDF]2.0.CO;2.

Geist, H. J., and E. F. Lambin. 2004. Dynamic causal patterns of desertification. *BioScience* 54: 817. doi:10.1641/0006-3568(2004)054[0817:DCPOD]2.0.CO;2.

Gerstner, K., C. F. Dormann, A. Stein, A. M. Manceur, and R. Seppelt. 2014. Effects of land use on plant diversity - A global meta-analysis. *Journal of Applied Ecology* 51: 1690–1700. doi:10.1111/1365-2664.12329.

Gibson, L., T. M. Lee, L. P. Koh, B. W. Brook, T. A. Gardner, J. Barlow, C. A. Peres, C. J. A. Bradshaw, et al. 2011. Primary forests are irreplaceable for sustaining tropical biodiversity. *Nature* 478: 378–81. doi:10.1038/nature10425.

Govaerts, B., N. Verhulst, A. Castellanos-Navarrete, K. D. Sayre, J. Dixon, and L. Dendooven. 2009. Conservation agriculture and soil carbon sequestration: between myth and farmer reality. *Critical Reviews in Plant Sciences* 28: 97–122. doi:10.1080/07352680902776358.

Gray, M. A., S. L. Baldauf, P. J. Mayhew, and J. K. Hill. 2007. The response of avian feeding guilds to tropical forest disturbance. *Conservation biology : the journal of the Society for Conservation Biology* 21: 133–41. doi:10.1111/j.1523-1739.2006.00557.x.

Guo, L. B., and R. M. Gifford. 2002. Soil carbon stocks and land use change: a meta analysis. *Global Change Biology* 8: 345–360. doi:10.1046/j.1354-1013.2002.00486.x.

Hadley, A. S., and M. G. Betts. 2012. The effects of landscape fragmentation on pollination dynamics: absence of evidence not evidence of absence. *Biological reviews of the Cambridge Philosophical Society* 87: 526–44. doi:10.1111/j.1469-185X.2011.00205.x.

Hahs, A. K., M. J. McDonnell, M. A. McCarthy, P. A. Vesk, R. T. Corlett, B. A. Norton, S. E. Clemants, R. P. Duncan, et al. 2009. A global synthesis of plant extinction rates in urban areas. *Ecology letters* 12: 1165–73. doi:10.1111/j.1461-0248.2009.01372.x.

Hergoualc'h, K. A., and L. V. Verchot. 2012. Changes in soil CH 4 fluxes from the conversion of tropical peat swamp forests: a meta-analysis. *Journal of Integrative Environmental Sciences* 9: 93–101. doi:10.1080/1943815X.2012.679282.

Hill, J. K., and K. C. Hamer. 2004. Determining impacts of habitat modification on diversity of tropical forest fauna: the importance of spatial scale. *Journal of Applied Ecology* 41: 744–754. doi:10.1111/j.0021-8901.2004.00926.x.

Holloway, G. L., and W. P. Smith. 2011. A meta-analysis of forest age and structure effects on northern flying squirrel densities. *The Journal of Wildlife Management* 75: 668–674. doi:10.1002/jwmg.77.

Hoogmoed, M., S. C. Cunningham, J. R. Thomson, P. J. Baker, J. Beringer, and T. R. Cavagnaro. 2012. Does afforestation of pastures increase sequestration of soil carbon in Mediterranean climates? *Agriculture, Ecosystems & Environment* 159: 176–183. doi:10.1016/j.agee.2012.07.011.

Huang, S., Y. Sun, and W. Zhang. 2011. Changes in soil organic carbon stocks as affected by cropping systems and cropping duration in China's paddy fields: a meta-analysis. *Climatic Change* 112: 847–858. doi:10.1007/s10584-011-0255-x.

Ilstedt, U., A. Malmer, E. Verbeeten, and D. Murdiyarso. 2007. The effect of afforestation on water infiltration in the tropics: A systematic review and meta-analysis. *Forest Ecology and Management* 251: 45–51. doi:10.1016/j.foreco.2007.06.014.

Iverson, A. L., L. E. Marín, K. K. Ennis, D. J. Gonthier, B. T. Connor-Barrie, J. L. Remfert, B. J. Cardinale, and I. Perfecto. 2014. REVIEW: Do polycultures promote win-wins or trade-offs in agricultural ecosystem services? A meta-analysis. *Journal of Applied Ecology* 51: 1593–1602. doi:10.1111/1365-2664.12334.

Jarecki, M. K., and R. Lal. 2003. Crop Management for Soil Carbon Sequestration. *Critical Reviews in Plant Sciences*. doi:10.1080/713608318.

Karthik, T., G. G. Veeraswami, and P. K. Samal. 2009. Forest recovery following shifting cultivation: an overview of existing research. *Tropical Conservation Science* 2: 374–387.
Kauffman, J. B., R. F. Hughes, and C. Heider. 2009. Carbon pool and biomass dynamics associated with deforestation, land use, and agricultural abandonment in the neotropics. *Ecological Applications* 19: 1211–1222. doi:10.1890/08-1696.1.
Keatley, B. E., E. M. Bennett, G. K. MacDonald, Z. E. Taranu, and I. Gregory-Eaves. 2011. Land-use legacies are important determinants of lake eutrophication in the anthropocene. *PloS one* 6: e15913. doi:10.1371/journal.pone.0015913.
Kennedy, C. M., E. Lonsdorf, M. C. Neel, N. M. Williams, T. H. Ricketts, R. Winfree, R. Bommarco, C. Brittain, et al. 2013. A global quantitative synthesis of local and landscape effects on wild bee pollinators in agroecosystems. *Ecology Letters* 16: 584–599. doi:10.1111/ele.12082.
Keys, E., and W. J. McConnell. 2005. Global change and the intensification of agriculture in the tropics. *Global Environmental Change* 15: 320–337. doi:10.1016/j.gloenvcha.2005.04.004.
Kim, D. G., D. Giltrap, and G. Hernandez-Ramirez. 2013. Background nitrous oxide emissions in agricultural and natural lands: A meta-analysis. *Plant and Soil* 373: 17–30. doi:10.1007/s11104-013-1762-5.
Kim, J. H., and R. B. Jackson. 2012. A global analysis of groundwater recharge for vegetation, climate, and soils. *Vadose Zone Journal* 11. doi:10.2136/vzj2011.0021RA.
Kleijn, D., and W. J. Sutherland. 2003. How effective are European agri-environment schemes in conserving and promoting biodiversity? *Journal of Applied Ecology* 40: 947–969. doi:10.1111/j.1365-2664.2003.00868.x.
Koh, L. P. . 2007. Impacts of land use change on South-east Asian forest butterflies: a review. *Journal of Applied Ecology* 44: 703–713. doi:10.1111/j.1365-2664.2007.01324.x.
Van Kooten, G. C., A. J. Eagle, J. Manley, and T. Smolak. 2004. How costly are carbon offsets? A meta-analysis of carbon forest sinks. *Environmental Science & Policy* 7: 239–251. doi:10.1016/j.envsci.2004.05.006.
Laliberté, E., J. A. Wells, F. Declerck, D. J. Metcalfe, C. P. Catterall, C. Queiroz, I. Aubin, S. P. Bonser, et al. 2010. Land-use intensification reduces functional redundancy and response diversity in plant communities. *Ecology letters* 13: 76–86. doi:10.1111/j.1461-0248.2009.01403.x.
Lawrence, D., C. Radel, K. Tully, B. Schmook, and L. Schneider. 2010. Untangling a decline in tropical forest resilience: constraints on the sustainability of shifting cultivation across the globe. *Biotropica* 42: 21–30. doi:10.1111/j.1744-7429.2009.00599.x.
Letourneau, D. K., I. Armbrecht, B. S. Rivera, J. M. Lerma, E. J. Carmona, M. C. Daza, S. Escobar, V. Galindo, et al. 2011. Does plant diversity benefit agroecosystems? A synthetic review. *Ecological Applications* 21: 9–21. doi:10.1890/09-2026.1.
Li, D., S. Niu, and Y. Luo. 2012. Global patterns of the dynamics of soil carbon and nitrogen stocks following afforestation: a meta-analysis. *The New Phytologist* 195: 172–81. doi:10.1111/j.1469-8137.2012.04150.x.
Liao, C., Y. Luo, C. Fang, J. Chen, and B. Li. 2012. The effects of plantation practice on soil properties based on the comparison between natural and planted forests: a meta-analysis. *Global Ecology and Biogeography* 21: 318–327. doi:10.1111/j.1466-8238.2011.00690.x.
Lu, Y., L. Chen, and B. Fu. 2008. Land-cover effects on red soil rehabilitation in China: a meta-analysis. *Progress in Physical Geography* 32: 491–502. doi:10.1177/0309133308098942.
Luck, G. W. 2007. A review of the relationships between human population density and biodiversity. *Biological reviews of the Cambridge Philosophical Society* 82: 607–45. doi:10.1111/j.1469-185X.2007.00028.x.
MacDonald, G. K., E. M. Bennett, and Z. E. Taranu. 2012. The influence of time, soil characteristics, and land-use history on soil phosphorus legacies: a global meta-analysis. *Global Change Biology* 18: 1904–1917. doi:10.1111/j.1365-2486.2012.02653.x.
Maetens, W., M. Vanmaercke, J. Poesen, B. Jankauskas, G. Jankauskiene, and I. Ionita. 2012. Effects of land use on annual runoff and soil loss in Europe and the Mediterranean: A meta-analysis of plot data. *Progress in Physical Geography*. doi:10.1177/0309133312451303.
Magliocca, N. R., T. K. Rudel, P. H. Verburg, W. J. McConnell, O. Mertz, K. Gerstner, A. Heinimann, and E. C. Ellis. 2015. Synthesis in land change science: methodological patterns, challenges, and guidelines. *Regional Environmental Change* 15: 211–226. doi:10.1007/s10113-014-0626-8.
Mantyka-pringle, C. S., T. G. Martin, and J. R. Rhodes. 2012. Interactions between climate and habitat loss effects on biodiversity: a systematic review and meta-analysis. *Global Change Biology* 18: 1239–1252. doi:10.1111/j.1365-2486.2011.02593.x.
Marczak, L. B., T. Sakamaki, S. L. Turvey, I. Deguise, S. L. R. Wood, and J. S. Richardson. 2010. Are forested buffers an effective conservation strategy for riparian fauna? An assessment using meta-analysis. *Ecological Applications* 20: 126–134. doi:10.1890/08-2064.1.
Marín-Spiotta, E., and S. Sharma. 2013. Carbon storage in successional and plantation forest soils: A tropical analysis. *Global Ecology and Biogeography*. doi:10.1111/j.1466-8238.2012.00788.x.
Martinson, H. M., and M. J. Raupp. 2013. A meta-analysis of the effects of urbanization on ground beetle communities. *Ecosphere* 4: art60. doi:10.1890/ES12-00262.1.

Meyfroidt, P., K. M. Carlson, M. E. Fagan, V. H. Gutiérrez-Vélez, M. N. Macedo, L. M. Curran, R. S. DeFries, G. A. Dyer, et al. 2014. Multiple pathways of commodity crop expansion in tropical forest landscapes. *Environmental Research Letters* 9: 074012. doi:10.1088/1748-9326/9/7/074012.

Misselhorn, A. A. 2005. What drives food insecurity in southern Africa? a meta-analysis of household economy studies. *Global Environmental Change* 15: 33–43. doi:10.1016/j.gloenvcha.2004.11.003.

Mondal, P., and H. Nagendra. 2011. Trends of forest dynamics in tiger landscapes across Asia. *Environmental management* 48: 781–94. doi:10.1007/s00267-011-9720-6.

Montero-Castaño, A., and M. Vilà. 2012. Impact of landscape alteration and invasions on pollinators: a meta-analysis. *Journal of Ecology* 100: 884–893. doi:10.1111/j.1365-2745.2012.01968.x.

Mori, A. S., and R. Kitagawa. 2014. Retention forestry as a major paradigm for safeguarding forest biodiversity in productive landscapes: A global meta-analysis. *Biological Conservation* 175: 65–73. doi:10.1016/j.biocon.2014.04.016.

Muchena, F. N., D. D. Onduru, G. N. Gachini, and A. de Jager. 2005. Turning the tides of soil degradation in Africa: capturing the reality and exploring opportunities. *Land Use Policy* 22: 23–31. doi:10.1016/j.landusepol.2003.07.001.

Munteanu, C., T. Kuemmerle, M. Boltiziar, V. Butsic, U. Gimmi, D. Kaim, G. Király, É. Konkoly-Gyuró, et al. 2014. Forest and agricultural land change in the Carpathian region—A meta-analysis of long-term patterns and drivers of change. *Land Use Policy*. doi:10.1016/j.landusepol.2014.01.012.

Murphy, G. E. P., and T. N. Romanuk. 2014. A meta-analysis of declines in local species richness from human disturbances. *Ecology and evolution* 4: 91–103. doi:10.1002/ece3.909.

Nichols, E., T. Larsen, S. Spector, A. L. Davis, F. Escobar, M. Favila, and K. Vulinec. 2007. Global dung beetle response to tropical forest modification and fragmentation: A quantitative literature review and meta-analysis. *Biological Conservation* 137: 1–19. doi:10.1016/j.biocon.2007.01.023.

Ogle, S. M., F. J. Breidt, and K. Paustian. 2005. Agricultural management impacts on soil organic carbon storage under moist and dry climatic conditions of temperate and tropical regions. *Biogeochemistry* 72: 87–121. doi:10.1007/s10533-004-0360-2.

Ogle, S. M., A. Swan, and K. Paustian. 2012. No-till management impacts on crop productivity, carbon input and soil carbon sequestration. *Agriculture, Ecosystems & Environment* 149: 37–49. doi:10.1016/j.agee.2011.12.010.

Pagdee, A., Y. Kim, and P. J. Daugherty. 2006. What makes community forest management successful: A meta-study from community forests throughout the world. *Society & Natural Resources* 19: 33–52. doi:10.1080/08941920500323260.

Paillet, Y., L. Bergès, J. Hjältén, P. Odor, C. Avon, M. Bernhardt-Römermann, R.-J. Bijlsma, L. De Bruyn, et al. 2010. Biodiversity differences between managed and unmanaged forests: meta-analysis of species richness in Europe. *Conservation biology : the journal of the Society for Conservation Biology* 24: 101–12. doi:10.1111/j.1523-1739.2009.01399.x.

Petheram, C., G. Walker, R. Grayson, T. Thierfelder, and L. Zhang. 2002. Towards a framework for predicting impacts of land-use on recharge: 1. A review of recharge studies in Australia. *Australian Journal of Soil Research* 40: 397. doi:10.1071/SR00057.

Philpott, S. M., W. J. Arendt, I. Armbrecht, P. Bichier, T. V Diestch, C. Gordon, R. Greenberg, I. Perfecto, et al. 2008. Biodiversity loss in Latin American coffee landscapes: review of the evidence on ants, birds, and trees. *Conservation biology : the journal of the Society for Conservation Biology* 22: 1093–1105. doi:10.1111/j.1523-1739.2008.01029.x.

Poeplau, C., A. Don, L. Vesterdal, J. Leifeld, B. Van Wesemael, J. Schumacher, and A. Gensior. 2011. Temporal dynamics of soil organic carbon after land-use change in the temperate zone - carbon response functions as a model approach. *Global Change Biology* 17: 2415–2427. doi:10.1111/j.1365-2486.2011.02408.x.

Ponisio, L. C., L. K. M'Gonigle, K. C. Mace, J. Palomino, P. de Valpine, and C. Kremen. 2014. Diversification practices reduce organic to conventional yield gap. *Proceedings of the Royal Society B: Biological Sciences*. doi:10.1098/rspb.2014.1396.

De Ponti, T., B. Rijk, and M. K. Van Ittersum. 2012. The crop yield gap between organic and conventional agriculture. *Agricultural Systems* 108: 1–9. doi:10.1016/j.agsy.2011.12.004.

Powers, J. S., M. D. Corre, T. E. Twine, and E. Veldkamp. 2011. Geographic bias of field observations of soil carbon stocks with tropical land-use changes precludes spatial extrapolation. *Proceedings of the National Academy of Sciences of the United States of America* 108: 6318–22. doi:10.1073/pnas.1016774108.

Prieto-Benítez, S., and M. Méndez. 2011. Effects of land management on the abundance and richness of spiders (Araneae): A meta-analysis. *Biological Conservation* 144: 683–691. doi:10.1016/j.biocon.2010.11.024.

Putz, F. E., P. A. Zuidema, T. Synnott, M. Peña-Claros, M. A. Pinard, D. Sheil, J. K. Vanclay, P. Sist, et al. 2012. Sustaining conservation values in selectively logged tropical forests: the attained and the attainable. *Conservation Letters* 5: 296–303. doi:10.1111/j.1755-263X.2012.00242.x.

Queiroz, C., R. Beilin, C. Folke, and R. Lindborg. 2014. Farmland abandonment: threat or opportunity for biodiversity conservation? A global review. *Frontiers in Ecology and the Environment* 12: 288–296. doi:10.1890/120348.

Rahmann. 2011. Biodiversity and organic farming: what do we know? *Landbauforschung* 61: 189–208.

Ramírez, P. A., and J. A. Simonetti. 2011. Conservation opportunities in commercial plantations: The case of mammals. *Journal for Nature Conservation* 19: 351–355. doi:10.1016/j.jnc.2011.06.003.

Rey Benayas, J. M., A. Martins, J. M. Nicolau, and J. J. Schulz. 2007. Abandonment of agricultural land: an overview of drivers and consequences. *CAB reviews: Perspectives in Agriculture Veterinary Science, Nutrition and Natural Resources* 2. doi:10.1079/PAVSNNR20072057.

Rey Benayas, J. M., A. C. Newton, A. Diaz, and J. M. Bullock. 2009. Enhancement of biodiversity and ecosystem services by ecological restoration: a meta-analysis. *Science (New York, N.Y.)* 325: 1121–4. doi:10.1126/science.1172460.

Richardson, J. S., and S. Béraud. 2014. Effects of riparian forest harvest on streams: a meta-analysis. *Journal of Applied Ecology*: n/a–n/a. doi:10.1111/1365-2664.12332.

Rindfuss, R. R., B. Entwisle, S. J. Walsh, C. F. Mena, C. M. Erlien, and C. L. Gray. 2007. Frontier Land Use Change: Synthesis, Challenges, and Next Steps. *Annals of the Association of American Geographers* 97: 739–754. doi:10.1111/j.1467-8306.2007.00580.x.

Rivest, D., A. Paquette, G. Moreno, and C. Messier. 2013. A meta-analysis reveals mostly neutral influence of scattered trees on pasture yield along with some contrasted effects depending on functional groups and rainfall conditions. *Agriculture, Ecosystems and Environment* 165: 74–79. doi:10.1016/j.agee.2012.12.010.

Robinson, B. E., M. B. Holland, and L. Naughton-Treves. 2014. Does secure land tenure save forests? A meta-analysis of the relationship between land tenure and tropical deforestation. *Global Environmental Change* 29: 281–293. doi:10.1016/j.gloenvcha.2013.05.012.

Rudel, T. K. 2007. Changing agents of deforestation: From state-initiated to enterprise driven processes, 1970–2000. *Land Use Policy* 24: 35–41. doi:10.1016/j.landusepol.2005.11.004.

Rudel, T. K., R. Defries, G. P. Asner, and W. F. Laurance. 2009. Changing drivers of deforestation and new opportunities for conservation. *Conservation biology : the Journal of the Society for Conservation Biology* 23: 1396–405. doi:10.1111/j.1523-1739.2009.01332.x.

Rusinamhodzi, L., M. Corbeels, M. T. Van Wijk, M. C. Rufino, J. Nyamangara, and K. E. Giller. 2011. A meta-analysis of long-term effects of conservation agriculture on maize grain yield under rain-fed conditions. *Agronomy for Sustainable Development*. doi:10.1007/s13593-011-0040-2.

Rutte, C. 2011. The sacred commons: Conflicts and solutions of resource management in sacred natural sites. *Biological Conservation* 144: 2387–2394. doi:10.1016/j.biocon.2011.06.017.

Salemi, L. F., J. D. Groppo, R. Trevisan, J. Marcos de Moraes, W. de Paula Lima, and L. A. Martinelli. 2012. Riparian vegetation and water yield: A synthesis. *Journal of Hydrology*. doi:10.1016/j.jhydrol.2012.05.061.

Scanlon, B. R., K. E. Keese, A. L. Flint, L. E. Flint, C. B. Gaye, W. M. Edmunds, and I. Simmers. 2006. Global synthesis of groundwater recharge in semiarid and arid regions. *Hydrological Processes* 20: 3335–3370. doi:10.1002/hyp.6335.

Schlossberg, S., and D. I. King. 2009. Postlogging succession and habitat usage of shrubland birds. *Journal of Wildlife Management* 73: 226–231. doi:10.2193/2007-518.

Schmidt, I. B., L. Mandle, T. Ticktin, and O. G. Gaoue. 2011. What do matrix population models reveal about the sustainability of non-timber forest product harvest? *Journal of Applied Ecology* 48: 815–826. doi:10.1111/j.1365-2664.2011.01999.x.

Schmidt-Vogt, D., S. J. Leisz, O. Mertz, A. Heinimann, T. Thiha, P. Messerli, M. Epprecht, P. Van Cu, et al. 2009. An assessment of trends in the extent of swidden in Southeast Asia. *Human Ecology* 37: 269–280. doi:10.1007/s10745-009-9239-0.

Schueler, T. R., L. Fraley-McNeal, and K. Cappiella. 2009. Is impervious cover still important? review of recent research. *Journal of Hydrologic Engineering* 14: 309–315. doi:10.1061/(ASCE)1084-0699(2009)14:4(309).

Seto, K. C., M. Fragkias, B. Güneralp, and M. K. Reilly. 2011. A meta-analysis of global urban land expansion. *PloS one* 6: e23777. doi:10.1371/journal.pone.0023777.

Seufert, V., N. Ramankutty, and J. A. Foley. 2012. Comparing the yields of organic and conventional agriculture. *Nature*. doi:10.1038/nature11069.

Shi, S., W. Zhang, P. Zhang, Y. Yu, and F. Ding. 2013. A synthesis of change in deep soil organic carbon stores with afforestation of agricultural soils. *Forest Ecology and Management* 296: 53–63. doi:10.1016/j.foreco.2013.01.026.

Skinner, C., A. Gattinger, A. Muller, P. Mäder, A. Fliebach, M. Stolze, R. Ruser, and U. Niggli. 2014. Greenhouse gas fluxes from agricultural soils under organic and non-organic management - A global meta-analysis. *Science of the Total Environment* 468-469: 553–563. doi:10.1016/j.scitotenv.2013.08.098.

Smucker, N. J., and N. E. Detenbeck. 2014. Meta-Analysis of Lost Ecosystem Attributes in Urban Streams and the Effectiveness of Out-of-Channel Management Practices. *Restoration Ecology*. doi:10.1111/rec.12134.

Sodhi, N. S., T. M. Lee, L. P. Koh, and B. W. Brook. 2009. A meta-analysis of the impact of anthropogenic forest disturbance on Southeast Asia's biotas. *Biotropica* 41: 103–109. doi:10.1111/j.1744-7429.2008.00460.x.

Srinivasan, V., E. F. Lambin, S. M. Gorelick, B. H. Thompson, and S. Rozelle. 2012. The nature and causes of the global water crisis: Syndromes from a meta-analysis of coupled human-water studies. *Water Resources Research* 48: n/a–n/a. doi:10.1029/2011WR011087.

Stewart, G. B., A. S. Pullin, and C. F. Coles. 2007. Poor evidence-base for assessment of windfarm impacts on birds. *Environmental Conservation* 34: 1. doi:10.1017/S0376892907003554.

Taranu, Z. E., and I. Gregory-Eaves. 2008. Quantifying relationships among phosphorus, agriculture, and lake depth at an inter-regional scale. *Ecosystems* 11: 715–725. doi:10.1007/s10021-008-9153-0.

Ticktin, T. 2004. The ecological implications of harvesting non-timber forest products. *Journal of Applied Ecology* 41: 11–21. doi:10.1111/j.1365-2664.2004.00859.x.
Tonitto, C., M. B. David, and L. E. Drinkwater. 2006. Replacing bare fallows with cover crops in fertilizer-intensive cropping systems: A meta-analysis of crop yield and N dynamics. *Agriculture, Ecosystems & Environment* 112: 58–72. doi:10.1016/j.agee.2005.07.003.
Tuck, S. L., C. Winqvist, F. Mota, J. Ahnström, L. A. Turnbull, and J. Bengtsson. 2014. Land-use intensity and the effects of organic farming on biodiversity: A hierarchical meta-analysis. *Journal of Applied Ecology*. doi:10.1111/1365-2664.12219.
Tuomisto, H. L., I. D. Hodge, P. Riordan, and D. W. Macdonald. 2012. Does organic farming reduce environmental impacts? - A meta-analysis of European research. *Journal of Environmental Management* 112: 309–320. doi:10.1016/j.jenvman.2012.08.018.
Tylianakis, J. M., R. K. Didham, J. Bascompte, and D. A. Wardle. 2008. Global change and species interactions in terrestrial ecosystems. *Ecology Letters* 11: 1351–1363. doi:10.1111/j.1461-0248.2008.01250.x.
Vanderwel, M. C., J. R. Malcolm, and S. C. Mills. 2007. A meta-analysis of bird responses to uniform partial harvesting across North America. *Conservation biology : the journal of the Society for Conservation Biology* 21: 1230–40. doi:10.1111/j.1523-1739.2007.00756.x.
Vellend, M., K. Verheyen, K. M. Flinn, H. Jacquemyn, A. Kolb, H. Van Claster, G. Peterken, B. J. Graae, et al. 2007. Homogenization of forest plant communities and weakening of species?environment relationships via agricultural land use. *Journal of Ecology* 95: 565–573. doi:10.1111/j.1365-2745.2007.01233.x.
Van Vliet, J., H. L. F. de Groot, P. Rietveld, and P. H. Verburg. 2015. Manifestations and underlying drivers of agricultural land use change in Europe. *Landscape and Urban Planning* 133: 24–36. doi:10.1016/j.landurbplan.2014.09.001.
Van Vliet, N., O. Mertz, A. Heinimann, T. Langanke, U. Pascual, B. Schmook, C. Adams, D. Schmidt-Vogt, et al. 2012. Trends, drivers and impacts of changes in swidden cultivation in tropical forest-agriculture frontiers: A global assessment. *Global Environmental Change* 22: 418–429. doi:10.1016/j.gloenvcha.2011.10.009.
Van Vliet, N., A. Reenberg, and L. V. Rasmussen. 2013. Scientific documentation of crop land changes in the Sahel: A half empty box of knowledge to support policy? *Journal of Arid Environments* 95: 1–13. doi:10.1016/j.jaridenv.2013.03.010.
Wickham, J. D., T. G. Wade, and K. H. Riitters. 2008. Detecting temporal change in watershed nutrient yields. *Environmental management* 42: 223–31. doi:10.1007/s00267-008-9120-8.
Winfree, R., R. Aguilar, D. P. Vázquez, G. LeBuhn, and M. A. Aizen. 2009. A meta-analysis of bees' responses to anthropogenic disturbance. *Ecology* 90: 2068–2076. doi:10.1890/08-1245.1.
Winfree, R., I. Bartomeus, and D. P. Cariveau. 2011. Native pollinators in anthropogenic habitats. *Annual Review of Ecology, Evolution, and Systematics* 42: 1–22. doi:10.1146/annurev-ecolsys-102710-145042.
Wright, J. P., and J. D. Fridley. 2010. Biogeographic synthesis of secondary succession rates in eastern North America. *Journal of Biogeography* 37: 1584–1596. doi:10.1111/j.1365-2699.2010.02298.x.
Zhou, D., S. Q. Zhao, S. Liu, and J. Oeding. 2013. A meta-analysis on the impacts of partial cutting on forest structure and carbon storage. *Biogeosciences* 10: 3691–3703. doi:10.5194/bg-10-3691-2013.
Ziegler, A. D., J. Phelps, J. Q. Yuen, E. L. Webb, D. Lawrence, J. M. Fox, T. B. Bruun, S. J. Leisz, et al. 2012. Carbon outcomes of major land-cover transitions in SE Asia: great uncertainties and REDD+ policy implications. *Global Change Biology* 18: 3087–3099. doi:10.1111/j.1365-2486.2012.02747.x.
